# Clinical Practice Guidelines on the Diagnosis and Management of Polycystic Ovary Syndrome: A Systematic Review and Quality Assessment Study

**DOI:** 10.1210/clinem/dgab232

**Published:** 2021-04-11

**Authors:** Bassel H Al Wattar, Maria Fisher, Laura Bevington, Vikram Talaulikar, Melanie Davies, Gerrad Conway, Ephia Yasmin

**Affiliations:** 1 Warwick Medical School, University of Warwick, Coventry, UK; 2 Reproductive medicine unit, University College London Hospitals, London, UK; 3 University Hospital Coventry & Warwickshire, Coventry, UK; 4 UCL Institute for Women’s Health, University College London, London, England

**Keywords:** polycystic ovary syndrome, clinical CPGs, quality, AGREE tool, systematic review

## Abstract

**Context:**

Clinical practice guidelines (CPGs) are key instruments to implement the practice of evidence-based medicine. We aimed to evaluate the methodological quality and variations in CPGs recommendations on the diagnosis and management of polycystic ovary syndrome (PCOS).

**Evidence Acquisition:**

We searched MEDLINE, EMBASE, and CENTRAL until December 2020 for all evidence-based CPGs and consensus statements on PCOS. We extracted data in duplicate to map clinical recommendations across prespecified disease domains and assessed CPGs methodological quality of using the Appraisal of Guidelines, Research & Evaluation II tool.

**Evidence Synthesis:**

We included 13 PCOS CPGs published between 2007 and 2018. CPGs recommendations were mostly focused on screening for and managing metabolic disease (12/13, 92%), followed by cardiovascular risk assessment (10/13, 77%). Mental health (8/13, 62%) and diagnosis in adolescents (7/13, 54%) were the least reported domains. Most CPGs had a high quality for scope and purpose description (12/13, 92%) while stakeholder’s involvement and applicability of recommendations to clinical practice were appropriate in only 2 CPGs (2/13, 15%). We identified inconsistency in recommendations on PCOS diagnosis in adolescents, optimal lifestyle interventions, hirsutism and acne treatments, interventions to reduce the risk of ovarian hyperstimulation syndrome, the frequency and screening criteria for metabolic and cardiovascular disease, and optimal screening tools for mental health illness in women with PCOS.

**Conclusion:**

Current CPGs on the diagnosis and management of PCOS vary in their scope and methodological quality, which may hinder evidence translation into clinical practice. We identified disease domains with existing evidence gap to guide future research and guideline updates.

Polycystic ovary syndrome (PCOS) is the most common endocrine condition affecting women of reproductive age worldwide (1). It significantly impacts women’s well-being and quality of life often increasing the risk of long-term health complications such as subfertility, type 2 diabetes, metabolic syndrome, and endometrial cancer (2). The variation in PCOS phenotype and expressed symptoms across affected women often leads to delayed diagnosis and symptomatic rather than comprehensive evidence-based treatment plans (3). Harmonizing clinical care within multidisciplinary teams and incorporating patients’ health needs could help to optimize treatment plans and improve the long-term health outcomes (4).

Adopting the principles of evidence-based medicine (EBM) has stimulated research conduct and evidence synthesis on the diagnosis and management of PCOS over the past few decades (4). Still, PCOS research remained largely segregated within different specialist disciplines caring for affected women such as primary care, endocrinology, gynecology, etc, leading to poor integration of evidence and undesired heterogeneity in research conduct (5).

Clinical practice guidelines (CPGs) and consensus statements are now primary tools to enable the practice of EBM and facilitate the implementation of evidence in everyday clinical practice (6). Traditionally, CPGs were developed within professional societies and specialty health regulators. This, however, led to concerns about the CPG inclusiveness, scope, and applicability to address patients’ needs in real life (7). Specifically, engaging lay consumers in the process of guideline production could help to focus the scope of CPGs on patients’ health needs and optimize the adoption of CPG recommendations into clinical practice (8). Several quality standards and development frameworks were established to optimize CPG implementation in clinical practice, increase their relevance, and promote inclusiveness of key stakeholders in the process (9-11). Adopting these principles into PCOS CPGs could be particularly challenging given its varied presentation o, lifelong impact on affected women, and the numerous disciplines involved in PCOS care provision (4). We aimed to systematically review all available CPGs on the management of PCOS and assess their quality using the Appraisal of Guidelines, Research & Evaluation II (AGREE II) tool (10).

## Methods

We undertook a systematic review using a prospectively registered protocol (CRD42018116809) and reported in line with the PRISMA CPGs (12).

### Literature Search

We searched MEDLINE, EMBASE, and Cochrane CENTRAL databases within the NICE Healthcare Database Advanced Search platform (hdas.nice.org.uk) using the following search terms to identify eligible CPGs on PCOS from inception until December 2020: clinical, CPGs, consensus statement, position statement, recommendation?, polycystic ovary, polycystic ovary syndrome, hyperandrogen*, anovulation, *menorrhea, wom?n, female, pregnan*. Complementary searches were conducted in Google Scholar, Tripdatabase, and Scopus. We searched the websites of established regulatory bodies on the topic of care for women with PCOS to identify relevant CPGs. We did not apply any search filters or language limitations.

### Guideline Selection and Inclusion Process

Two independent reviewers (BHA and MF) completed the study selection and inclusion process in 2 stages. Discrepancies were resolved in consensus with a third reviewer (LB). We included all purpose-developed evidence-based clinical CPGs and consensus statements addressing the diagnosis and/or management of PCOS across different clinical disciplines and populations. We excluded position or opinion statements that did not make direct recommendations linked to specific evidence and systematic reviews from scientific societies. All other designs of primary or secondary studies evaluating a particular scientific question were excluded.

### Data Collection

Two independent reviewers (LB and MF) extracted data in duplicate into an electronic Excel sheet, which was piloted for its face validity. Data integrity was double-checked by a third reviewer (BHA). We extracted data on the following guideline characteristics: producing authority, named authors, country of origin, year of publication, consensus method, stakeholders involved, disease domain addressed in the CPG, description of the search strategy to identify evidence, inclusion/exclusion criteria of evidence, quality assessment instruments used, and grading system used. We mapped out the clinical recommendations in each guideline and tabulated them into the following prespecified domains: diagnosis in adolescents and adults; lifestyle interventions; management of menstrual irregularity, hirsutism, acne, and infertility; and risk assessment for metabolic disease, cardiovascular disease, mental health, and cancer.

### Assessment of Methodological Quality

We used the AGREE II instrument (10) to assess the methodological quality of each guideline in six domains (scope and purpose, stakeholder involvement, rigor of development, clarity and presentation, applicability, editorial independence) and 23 items. Each item was scored using a 7-point Likert scale anchored between 1 (strongly disagree) and 7 (strongly agree). We generated a total quality score using a prescribed formula (10). We categorized scores to offer a summative quality measure for each domain with scores from 10 to 7 showing high quality; 7 to 4, medium quality; and below 4, low quality of guideline development.

### Statistical Analysis

We calculated a total quality score for each guideline by adding the scores of the evaluated quality domains and standardizing them using a prescribed equation (10). We reported on descriptive data using normal frequencies, median, and ranges. We assessed correlation coefficients using Pearson correlation test. All statistical analyses were conducted using Microsoft Excel (Excel 2017, Microsoft, Redmond, WA, USA).

## Results

**Figure 1.**
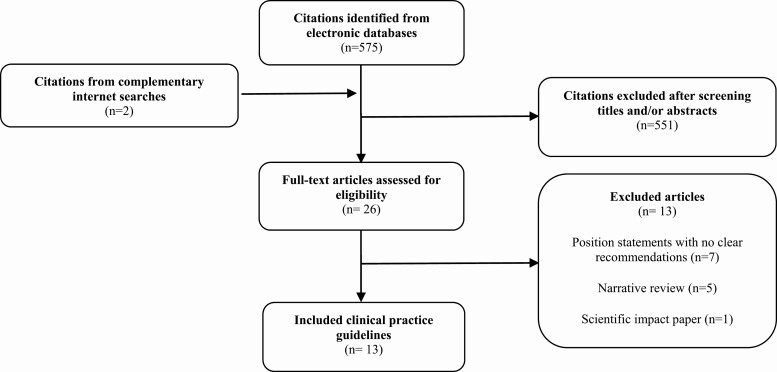
Selection and inclusion process for the systematic review on the quality of evidence-based clinical practice guidelines on polycystic ovarian syndrome.

Our electronic search identified 575 titles and abstracts of which we screened 26 articles in full against our inclusion criteria and included a total of 5 national and 8 international PCOS CPGs (n = 13) (Fig. 1).

The majority of included CPGs were published within the last 10 years (range 2007-2018) and all but 1 (13) were reported as peer-reviewed. Most CPGs used simple panel discussions to reach consensus among coauthors, and only 2 (2/13, 15%) used an established consensus methodology (eg, Delphi method) (14,15). Seven CPGs used a clear evidence grading system when making recommendations (7/13, 54%), and only 2 (2/13, 15%) provided clear implementation tools for evidence into clinical practice (14,15) (Table 1).

**Table 1.** Characteristics of included evidence-based clinical practice guidelines on polycystic ovary syndrome

Producing authority	Publication year	Peer reviewed	Consensus methodology	Search strategy	Inclusion/exclusion criteria	Evidence grading system	Implementation tools	Number of recommendations
ICPE (16)	2017	Yes	Panel discussion	N/A	N/A	N/A	No	26
AE-PCOS (17)	2010	Yes	Panel discussion	Systematic review of published peer-reviewed medical literature by 3 investigators.	Inclusion: CVD risk factors for women with and without PCOS.	N/A	No	13
					Exclusion: other hyperandrogenic disorders were not excluded, PCOS diagnosis uncertain, controls not described			
NHMRC (14)	2011	Yes	Consensus voting technique	An internet search strategy for evidence-based guidelines and systematic reviews using the Google ‘Advanced Search’ function	Included guidelines <4 years old, pass the AGREE benchmark criteria (Systematic methods used to search for evidence with an explicit link between the recommendations and the supporting evidence)	NHMRC	Yes	66
ES (18)	2013	Yes	Panel discussion	Systematic review of published literature	N/A	GRADE system	No	27
IFS (19)	2018	Yes	Panel discussion	Systematic review of existing guidelines, meta-analyses, systematic reviews, key cited articles	N/A	GRADE system	No	59
CREPCOS (13)	2018	Yes	Delphi and nominal group techniques	Systematic review	N/A	GRADE system	Yes	90 + 76 clinical practice points
AES (20)	2007	Yes	Panel discussion	Systematic review on MEDLINE	Excluded unpublished data or data published only in abstract for were not included	N/A	No	6
RANZCOG (21)	2017	Yes	N/A	Systematic review on MEDLINE	N/A	N/A	No	9
RCOG (22)	2014	Yes	Committee consensus	Systematic review	Inclusion: 'PCOS', 'metabolic', 'diabetes', 'cardiovascular', 'cancer', English language, limited to humans.	Green-top Grading	No	19
PES (23)	2015	Yes	Committee consensus	Systematic review	N/A	AGREE criteria	No	27
AACE (24)	2015	Yes	Committee consensus	Systematic review	N/A	N/A	No	11
ACOG (12)	2018	No	N/A	Systematic review MEDLINE database, the Cochrane Library, and ACOG’s own internal resources and documents	N/A	US preventive services task force	No	13
ESHRE/ASRM (25)	2008	Yes	Panel discussion	N/A	N/A	N/A	No	55

The median number of recommendations made per guideline was 26 (range 6-90). Screening for and managing metabolic disease was the most commonly covered disease domain in 12 CPGs (12/13, 92%) followed by recommendations on cardiovascular risk assessment (10/13, 77%). In contrast, management of mental health (8/13, 62%) and diagnosis in adolescents (7/13, 54%) were the least commonly addressed disease domains across the included CPGs (Table 2).

**Table 2.** Summary of disease domains covered by recommendations in evidence-based clinical practice guidelines on polycystic ovary syndrome

Guideline	Diagnosis in adolescents	Diagnosis in adults	Lifestyle	Menstrual irregularity	Hirsutism and acne	Infertility	Metabolic disease	Mental health	Cardiovascular disease
ICPE (16)	✓	x	✓	✓	✓	x	✓	✓	✓
AE-PCOS (17)	x	x	x	x	x	x	✓	✓	✓
NHMRC (14)	✓	✓	✓	✓	✓	✓	✓	✓	✓
ES (18)	✓	✓	✓	✓	✓	✓	✓	✓	✓
IFS (19)	✓	✓	✓	✓	✓	x	✓	✓	✓
CREPCOS (13)	✓	✓	✓	✓	✓	✓	✓	✓	✓
AES (20)	x	x	x	x	x	x	✓	x	✓
RANZCOG (21)	x	✓	✓	x	x	✓	✓	✓	✓
RCOG (22)	x	✓	✓	✓	✓	x	✓	✓	✓
PES (23)	✓	x	x	x	x	✓	x	x	x
AACE (24)	✓	✓	x	x	✓	x	✓	x	x
ACOG (12)	x	x	✓	✓	✓	✓	✓	x	✓
ESHRE/ASRM (25)	x	x	✓	x	x	✓	✓	x	x

✓, covered, x, not covered.

### Guideline Quality

**Figure 2.**
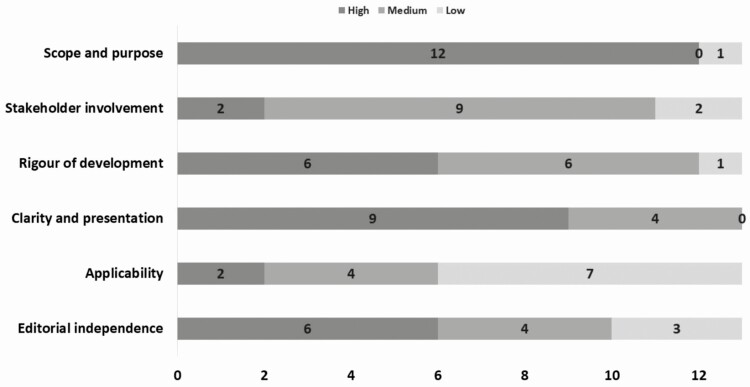
Quality of included evidence-based clinical practice guidelines on polycystic ovarian syndrome using the AGREE II tool. Clinical practice guidelines were evaluated for their development methodology and reporting in 23 items grouped into 6 domains using the AGREE II tool. Guidelines that scored in the top tertile were reported to have high quality for each of the domains.

Our evaluation of CPGs’ quality using the AGREE II tool showed variations across the assessed domains with most CPGs offering high-quality scope and purpose description (12/13, 92%) as well as clarity in presentation (9/13, 69%). Stakeholder involvement and applicability of recommendation to clinical practice were poorly addressed by most CPGs with only 2 showing good quality for these domains (2/13, 15%) (Fig. 2). There was a poor correlation between guideline quality and year of publication (r = −0.02).

### Summary of Recommendations

Majority of CPGs focused on the different treatments of PCOS (10/13, 77%), risk assessment (10/13, 77%), and only 9 made recommendations on the diagnosis of PCOS (9/13, 69%) (26).

### Diagnosis of PCOS

For the diagnosis of PCOS in adults, 7 CPGs supported the use of the Rotterdam criteria (24) or its modification (16), although only 1 provided a clear definition for of polycystic ovarian morphology (PCOM) in adults (14). Only 4 CPGs recommended systematic examination and assessment of clinical hyperandrogenism with only 1 guideline recommending the use of specific standardized assessment tools (Ferriman Gallwey score and the Ludwig visual score) for hirsutism and acne in adults (14). All relevant CPGs supported the use of testosterone (total or free) or the free androgen index (calculated as the ratio is the level of total testosterone divided by the level of sex hormone-binding globulin and multiplied by 100) to diagnose hyperandrogenemia, although there were variations on the value of androstenedione and dehydroepiandrosterone sulphate as routine blood tests to diagnose PCOS. Anti-Müllerian hormone was recommended as useful for diagnosing PCOS in 1 guideline (23) while the international PCOS guideline did not recommend its use (14).

Two CPGs were particularly focused on the diagnosis and management of PCOS in adolescents (18,19), and 5 generated recommendations on both adolescent and adult women with PCOS (14,15,21,23,25). All relevant CPGs recommended against the use of PCOM as an independent ultrasonic diagnostic feature in adolescents emphasizing the limited value of ultrasound scanning in this population. Only 2 CPGs made clear recommendations on the definitions for oligo and amenorrhea in adolescents as part of the PCOS diagnostic criteria (14,18). There were variations in the recommendations made on the value of different biochemical tests to diagnose hyperandrogenemia although all included CPGs recommended against the use of anti-Müllerian hormone.

### Lifestyle Interventions

Nine CPGs recommended lifestyle treatments (LSTs) as first-line intervention in adolescents and adult women with PCOS (13-15,17,19-21,25,27,28). The majority of these CPGs recommended a mixture of calorie-restricted diet, exercise, and behavioral interventions as the main features of LSTs. Four CPGs recommended a weight loss target between 5% and 10% with LSTs (15,19,25,27). There were no clear recommendations on the type of diet to offer women with PCOS with varied recommendations for hypocaloric diet (deficit between 500 and 700 kcal/day) and a focus on low glycemic index food intake (14,27). Similarly, there was no clear consensus on the optimal duration or type of physical exercise to recommend. Three CPGs recommended 150 min/week (14,15,25) and 90 min/week of aerobic moderate-high intensity exercise for weight maintenance. Three CPGs recommended combining LSTs with pharmacological treatments such as metformin to optimize weight loss after 6 months (13,19,20), and 3 recommended bariatric surgery in obese women with PCOS if LST alone failed to achieve sufficient weight loss (14,15,20).

### Management of Menstrual Irregularity

Six CPGs recommended the use of hormonal contraceptives as first-line of treatment for managing menstrual irregularity in adult women with PCOS (13-15,19,21,25) and 5 recommended their use in adolescents with suspected/confirmed PCOS diagnosis (14,15,19,21,25). The use of metformin, cyproterone acetate, and drospirenone was recommended as a second-line treatment for menstrual irregularity, hirsutism, and acne in 3 CPGs (14,21,25). Most of these CPGs recommended using the same screening criteria as for safe use of hormonal contraceptive in the general population. The use of progesterone to induce regular withdrawal bleeds in amenorrheic women with PCOS was recommended in 3 CPGs (13,19,20).

### Management of Hirsutism and Acne

Only 5 CPGs made direct recommendations on treatments for hirsutism and acne with 3 CPGs recommending photoepilation and topical eflornithine as the first line of treatment (13,19,25). By contrast, the Endocrine Society guideline recommended using hormonal contraceptives as the first-line treatment in adolescents with suspected PCOS to treat acne, hirsutism, and anovulatory symptoms. Antiandrogen medications alone or combined with hormonal contraceptives were recommended as second-line treatments in 4 CPGs (14,19,23,25).

### Management of Infertility

Overall LSTs were considered the optimal first-line treatment for anovulation in women with PCOS and infertility for 3 to 6 months (14,15,27). The Royal Australian and New Zealand College of Obstetricians and Gynaecologists guideline considered pharmacological ovulation induction a contraindication in obese women (body mass index > 35 kg/m^2^) with PCOS (20). Three CPGs recommended the use of letrozole as the primary method of pharmacological ovulation induction (13,14,21). This represented a shift in evidence compared to older CPGs such as the Australian National Health and Medical Research Council guideline, which considered it optional (15), and the Thessaloniki ESHRE/ASRM guideline which recommended clomiphene citrate as first-line treatment (27). Most CPGs recommended ovulation induction with gonadotropins or laparoscopic ovarian drilling as a second-line treatment. The Thessaloniki ESHRE/ASRM guideline recommended ovulation induction with a follicle stimulating hormone starting dose of 37.5 to 50 IU/day used up to a 14-day stimulation period with close ovulation monitoring and a maximum of 6 stimulation cycles (27). In vitro fertilization was suggested as last option with a preference for gonadotropin-releasing hormone antagonist protocols and metformin to reduce the risk of ovarian hyperstimulation syndrome (14,21), although there were limited recommendations on the best in vitro fertilization protocols in women with PCOS.

### Risk Assessment and Management of Metabolic Disease

Most of the included CPGs supported using metformin alone or in combination with other treatments (eg, hormonal contraceptives) in overweight/obese women to optimize weight management, reduce insulin resistance, and minimize hyperandrogenism (13-15,19,21,23,25,27,29,30). There was no advice regarding the point of commencement or duration of treatment. There was uncertainty on the safety of metformin in pregnancy especially in those who received it during ovulation induction. Two CPGs suggested stopping it once pregnancy is confirmed (14,25). Similarly, there were no clear criteria for its use in adolescents with suspected PCOS across included CPGs.

An oral glucose tolerance test was considered the gold standard to screen for impaired glucose intolerance and type 2 diabetes although there were variations on the recommended frequency of screening (Table 3). More frequent screening was suggested in women with risk factors for type 2 diabetes including body mass index > 25 kg/m^2^ or in Asians > 23 kg/m^2^, central adiposity, substantial weight gain, increased waist circumference, symptoms of diabetes, family history of impaired glucose intolerance, type 2 diabetes, chronic hypertension or high-risk ethnicity, age > 40 years, personal history of gestational diabetes or high blood glucose level, use of antihypertensive medications, smoking, and physical inactivity (13,14,17,18,28). Hemoglobin A1c was recommended as a substitute screening/diagnostic test when oral glucose tolerance test is not feasible (14,21,25).

**Table 3.** Summary of screening tools and risk assessment for women with polycystic ovary syndrome based suggested in evidence-based clinical practice guidelines

Disease domain	Screening tool	Suggested screening frequency
Impaired glucose tolerance and type 2 diabetes mellitus	Oral glucose tolerance test or hemoglobin A1c	No specific frequency, ranging from annually to once every 5 years, sooner if any of the following risk factors: body mass index >25 kg/m^2^ or in Asians >23 kg/m^2^; central adiposity; substantial weight gain; increased waist circumference; symptoms of diabetes; family history of impaired glucose intolerance, type 2 diabetes, or chronic hypertension; high-risk ethnicity; age >40 years; personal history of gestational diabetes or high blood glucose level; use of antihypertensive medications; smoking; physical inactivity
Cardiovascular disease	Screen for risk factors: obesity especially increased abdominal adiposity, smoking, hypertension, dyslipidemia, subclinical vascular disease, impaired glucose tolerance, family history of premature cardiovascular disease, physical inactivity, metabolic syndrome, type 2 diabetes, obstructive sleep apnea, high levels of CRP and homocysteine	No specific frequency
Hirsutism and Acne	Clinical assessment using standardized tools (Ferriman Gallwey score and the Ludwig visual score)	No specific frequency
Weight including waist circumference and body mass index	Direct measurement	Each visit with a minimum suggested period between 6 and 12 months
Blood pressure	Direct measurement	Each visit with a minimum suggested period between 6 and 12 months
Lipids	Serum blood tests	Every 2 years
Gestational diabetes	Oral glucose tolerance test	Between 24 and 28 weeks’ gestation
Obstructive sleep apnea	Clinical assessment of associated symptoms	Only in symptomatic women
Mental illness	PCOS quality of life tool (PCOSQ)	No specific frequency
Endometrial cancer	Transvaginal ultrasound scan to assess endometrial thickness	Only in women with unexpected uterine bleeding or spotting

Two CPGs recommended screening for nonalcoholic fatty liver disease and nonalcoholic steatohepatitis in women with insulin resistance and metabolic syndrome suggesting vitamin E as preferred treatment with specialist multidisciplinary team input (21,25).

### Mental Health

Routine assessment for mental health and quality of life was supported in seven CPGs (13,14,17-19,21,28) although only the international PCOS guideline recommended the use of specific screening and assessment tools (14). Referral for appropriate mental health counseling and management was recommended though no specific referral pathways were suggested (13,14,18,19,21,28).

### Cardiovascular Disease

Assessment of cardiovascular risk factors in women with PCOS was recommended in 8 CPGs using an array of risk factors including obesity especially increased abdominal adiposity, smoking, hypertension, dyslipidemia, subclinical vascular disease, impaired glucose tolerance, family history of premature cardiovascular disease, lack of physical activity, metabolic syndrome and type 2 diabetes, obstructive sleep apnea, high levels of CRP, and homocysteine (12-14,17-19,21,28). One guideline (25) supported the use of serum homocysteine as a test for hyperhomocysteinemia-mediated repeated pregnancy losses in women with previous miscarriage although the quality of associated evidence was poor.

Five CPGs recommended routine screening for obstructive sleep apnea in women with PCOS by checking associated symptoms such as snoring, waking unrefreshed from sleep, and daytime sleepiness. All these CPGs considered a polysomnography test as the gold standard diagnostic test for obstructive sleep apnea (13,18,19,21,28), and only the international PCOS guideline supported the use of the Berlin tool for screening in symptomatic women (14).

### Cancer

Routine screening for endometrial cancer was not recommended in women with PCOS in 3 CPGs (14,21,25). Two CPGs recommended performing a transvaginal ultrasound scan to evaluate the endometrium in case of abnormal uterine bleeding, spotting, and prolonged amenorrhea (>90 days) (14,21). Three CPGs recommended inducing a withdrawal bleeding using progesterone in women with prolonged amenorrhea every 3 to 4 months and offering an endometrial biopsy and/or hysteroscopy to assess thickened endometrium or an endometrial polyp (13,28) The Royal College of Obstetricians and Gynaecologists guideline considered hyperplasia to be unlikely in women with PCOS and an endometrial thickness <7 mm and also recommended no additional surveillance for breast or ovarian cancer (28).

## Discussion

### Summary of Main Findings

We captured a high number of evidence-based CPGs produced over the last decade covering varied disease domains with a consistently increasing number of recommendations. Guideline quality was varied with poor stakeholders involvement and a substantial lack of clear implementation pathways.

Reassuringly, most of the included CPGs recommended the use of homogenous PCOS diagnostic criteria. Still, there were variations in the recommended diagnostic reference ranges for biochemical hyperandrogenism and on the diagnostic value of anti-Müllerian hormone in adolescents with PCOS.

Most CPGs recommended routine screening for cardiovascular and metabolic diseases in affected women; however, the recommended frequency of screening, measurement tools, and associated risk factors varied substantially (Table 3). Mental health was particularly underrepresented in most of the included CPGs with much uncertainty on the most effective screening and treatment pathways for women with PCOS.

Most of the included CPGs emphasized the importance of LST as the first-line treatment strategy for PCOS, although details on specific dietary regimes were lacking. Similarly, the role of anti-obesity medications and insulin sensitizers was varied across included CPGs. Few CPGs recommended particular treatments for hyperandrogenism that could be linked to the limitations of available evidence on this domain. Lastly, with a growing body of evidence on fertility treatments for women with PCOS, there were clearer recommendations on the treatment pathways for anovulation and subfertility. Variations existed on the role of letrozole as the primary ovulation induction agent; however, this is unsurprising giving the evolution of evidence on its effectiveness and safety over the last decade. There was limited guidance about the optimal ovarian stimulation protocols and adjuvant treatments to reduce the risk of ovarian hyperstimulation syndrome in women with PCOS.

### Strength and Limitations

To our knowledge, our review is the first to evaluate the quality of all available evidence-based CPGs and consensus statements regarding the diagnosis and management of PCOS. We followed an established methodology to systematically review the literature and applied the AGREE II tool to assess guideline quality. Lastly, we mapped out CPGs recommendations to identify underrepresented disease domains and topics of uncertainty to aid future research conduct.

Our analysis has a few limitations. The use of the AGREE II tool is relatively recent and older CPGs may not have adopted recently developed standards, which may skew our findings. Specifically, the practice of involving stakeholders, including lay consumers, in CPGs development as well as active guideline implementation in clinical practice is relatively new and usually absent in older CPGs. We aimed to provide a systematic assessment of current literature to illustrate the improvement in guideline quality over time. Interestingly, we did not detect a correlation between guideline quality and year of publication.

We were unable to assess the quality and confidence in linked evidence to guideline recommendations as different evidence grading systems were used. This limited our ability to provide an in-depth analysis of the current evidence gap. The poor quality of available primary studies on PCOS is often reported as a source of heterogeneity and imprecision in published CPGs (14). Recognizing this evidence gap is helpful to specify future research need and priorities (4).

### Implications for Clinical Practice and Future Research

PCOS is uniquely expressed as a multisystemic condition often with varied phenotypes across affected women (31). To address the complexity of diagnosing and managing the different aspects of PCOS, care provision could be optimized within multidisciplinary teams shared across primary and secondary care (32). This vision, however, has not prevailed in most of the evaluated CPGs, many of which were produced by specialist professional societies focused on particular disease domains. Consequently, several important health aspects were infrequently present such as PCOS diagnosis in adolescents and screening for mental health issues. Going forward, future guideline updates should adopt the multidisciplinary approach to PCOS care provision to minimize fragmentation in health services and optimize women’s access to treatments (4). Specifically, involving lay consumers in future CPGs development could help the process of evidence implementation into clinical practice with a focus on addressing real patients’ health needs (22). For example, qualitative evidence suggested a common theme of women’s frustration on the delay in diagnosing PCOS (3). Acknowledging this need helped to focus research and policymaking efforts on developing clear diagnostic criteria for PCOS including specific diagnostic tools such as PCOM (14). Our findings suggest that more work is needed to investigate the role of promising diagnostic tests such as anti-Müllerian hormone and other biomarkers.

As more treatment options become available to address the different aspects of PCOS, there is a need to continuously update available CPGs as well as efficient data collection to enable large scale evidence synthesis and optimize the practice of EBM. This was particularly evident in this review with the limited scope of recommendations made on certain treatments such as the use of different contraceptives for the management of hirsutism and acne in PCOS (26). Similarly, several new pharmacological agents are now used as fertility treatments for women with PCOS, yet comprehensive and high-quality evidence to support their effectiveness remains limited (26). Addressing this evidence gap using randomized trials might be slow and inefficient given the varied phenotypes in women with PCOS. As such, investing in prospective large cohorts and big data research infrastructure may offer a valuable alternative to aid comprehensive evaluation of the effectiveness and safety of newly developed treatments (33).

## Conclusion

Current CPGs on the diagnosis and management of PCOS vary in their scope and methodological quality which may hinder evidence translation into clinical practice. We identified disease domains with existing evidence gap to guide future research and guideline updates.

## Data Availability

Some or all data sets generated during and/or analyzed during the current study are not publicly available but are available from the corresponding author on reasonable request.

## Additional Information

***Disclosures:*** The authors have nothing to disclose.
